# ∆^9^-Tetrahydrocannabinol Increases Growth Factor Release by Cultured Adipose Stem Cells and Adipose Tissue *in vivo*

**DOI:** 10.1007/s13770-024-00692-8

**Published:** 2025-01-18

**Authors:** Tim Ruhl, Sofija Benic, Melissa Plum, Bong-Sung Kim, Justus P. Beier, Benedikt Schaefer

**Affiliations:** 1 Department of Plastic Surgery, Hand Surgery–Burn Center, University Hospital RWTH Aachen, Pauwelsstraße 30, 52074 Aachen, Germany; 2 Present Address: Department of Plastic Surgery and Hand Surgery, University Hospital Zurich, Raemistrasse 100, 8091 Zurich, Switzerland

**Keywords:** Regenerative properties, Stroma cells, Cannabinoids

## Abstract

*****BACKGROUND***:**

Because of its biocompatibility and its soft and dynamic nature, the grafting of adipose tissue is regarded an ideal technique for soft-tissue repair. The adipose stem cells (ASCs) contribute significantly to the regenerative potential of adipose tissue, because they can differentiate into adipocytes and release growth factors for tissue repair and neovascularization to facilitate tissue survival. The present study tested the effect of administering a chronic low dose of ∆^9^-tetrahydrocannabinol (THC) on these regenerative properties, *in vitro* and *in vivo*.

*****METHODS***:**

Human ASCs were exposed to increasing concentrations of THC. Resazurin conversion was applied to investigate the effect on metabolic activity, cell number was assessed by crystal violet staining, tri-linear differentiation was evaluated by specific colorimetric approaches, and the release of growth factors was analyzed by ELISA. Two groups of mice were treated daily either with a low dose of THC (3 mg/kg) or a vehicle solution. After 3 weeks, adipose tissue was obtained from excised fat deposits, homogenized and tested for growth factor contents.

*****RESULTS***:**

THC decreased ASC proliferation but increased metabolic activity as well as adipogenic and chondrogenic differentiation. A low concentration of THC (1 µM) enhanced the growth factor release by ASCs. The concentration of these cytokines was also increased in adipose tissue of mice treated with THC.

*****CONLUSION***:**

Our results indicate that chronic activation of the endocannabinoid system promoted differentiation and growth factor release of ASCs, which could be of specific value for enhancing the regenerative potential of adipose tissue.

## Introduction

Adipose tissue holds various functions in maintenance of regular body functions, which includes energy storage, metabolic homeostasis, steroid production, immunoregulation, hematopoiesis, fertility and reproduction, as well as mechanical buffer to protect from external shocks [1, 2]. Especially because of its soft and dynamic nature and its easy harvest, adipose tissue has been used for decades to reconstruct soft-tissue deficits [3]. The discovery that adipose tissue contains not only adipocytes but also adipose stem or stromal cells (ASCs) significantly accelerated fat grafting as a regenerative therapy, since many of the benefits observed after lipofilling were attributed to ASC features [4]. Despite its widespread use, the main obstacle of grafting fat tissue is the unpredictable resorption, both in location and extent, which necessitates the need for repetitive procedures. Thus, various processing techniques have experimentally been evaluated to enhance the regenerative potential of adipose tissue in order to increase the chance for optimal outcome for graft retention, e.g. decantation, washing, centrifugation [5]. Although still an area of debate, enriching grafts with cells from the adipose stroma vascular fraction, which contains high amounts of ASCs, have been reported to increase graft viability and outcome after transplantation [6]. Actually two hypotheses on ASC action exist: Depending on their multipotent differentiation potential, ASCs could differentiate into mature adipocytes for replacement of the cells lost through apoptosis or necrosis. Alternatively, because of their secretory activity and especially the release of pro-angiogenic growth factors, e.g. vascular endothelial growth factor (VEGF) and hepatocyte growth factor (HGF), ASCs may promote neovascularization of transplanted adipose tissue [7].

Various experimental methods have been tested to advance the therapeutic effects of ASCs. In order to increase the release of trophic factors upon cell delivery *in vivo*, different approaches focus on genetic manipulation and in vitro priming or preconditioning of the cells [8–10]. However, as these techniques require the isolation, expansion, and sensitively the modification of ASCs, these processes represent substantial manipulations and fall, thus, under regulatory restrictions. As an alternate solution to the extracorporeal manipulation, ASCs could be primed at the donor site prior harvesting to increase their regenerative capability at the recipient site after grafting. The endocannabinoid system (ECS) is an endogenous instance for cellular and systemic maintenance of homeostatic processes [11]. We previously examined the expression and function of the ECS of ASCs in vitro and found that the cells express both the classical cannabinoid receptors, *i.e.*, CB1 and CB2, and the non-classical cannabinoid receptors, *e.g.* G-protein-coupled receptor (GPR)55 and transient receptor potential cation channel (TRPV)1 [12, 13]. Specific receptor stimulation of CB1 and CB2 promotes differentiation, especially into the adipogenic lineage, but it also increases the release of regenerative growth factors, including VEGF, HGF, and transforming growth factor (TGF)-β1 [14]. The same effects occur when ASCs are exposed to the endocannabinoids anandamide and 2‐arachidonoylglycerol [15]. Thus, we hypothesize that using a chronic low dose of medical cannabis to activate the ECS of ASCs prior fat grafting may be a possibility to positively modulate ASCs for regenerative approaches.

In the present study, we first confirmed our previous *in vitro* findings on ECS stimulation of ASCs. We investigated the effects of primary human ASC exposure to THC at increasing concentrations (1–5 μM) on viability and tri-linear differentiation. Furthermore, we measured the release of growth factors in the cell supernatants. We next performed the *in vivo* transfer, wherein mice received a chronic low dose of THC for a period of 3 weeks, before determination of growth factor levels in fully excised fat deposits.

## Material and methods

### Materials

Ascorbate 2-phosphate, β-glycerophosphate, paraformaldehyde (PFA), trypsin–EDTA, 2-amino-2-methyl-1-propanol (AMP), o-cresolphthalein complexon, 8-Hydroxyquinoline, Bovine serum albumin (BSA), Safranin O, Tween20, Kolliphor®P188 were obtained from Sigma (Taufkirchen, BY, Germany). L-Prolin, sodium-pyruvate, acetic acid, hydrochloric acid (HCl) and crystal violet were received from Roth (Karlsruhe, BW, Germany). Protease inhibitor cocktail, DAPI and insulin were bought from Roche (Mannheim, BW, Germany). Resazurin was purchased from Santa Cruz Biotechnology (Heidelberg, BW, Germany). Fetal bovine serum (FBS) was from Capricorn Scientific (Ebsdorfergrund, HE, Germany). ITS-G premix, high / low glucose medium (4.5 g/L) and Dulbecco’s Modified Eagle’s medium (DMEM/F-12) were obtained from Life Technologies (Darmstadt, HE, Germany). ∆^9^-tetrahydrocannabinol (Dronabinol) was bought from LGC (Wesel, NW, Germany). Collagenase (type I) was purchased from Worthington Biochemical Corp. (Lakewood, NJ, USA). Transforming growth factor-β3 (TGF-β3) was obtained from PeproTech (Hamburg, HH, Germany). 2-propanol and Oil Red O was bought from Merck (Darmstadt, HE, Germany).

### Cell culture

Fourteen healthy female Caucasian patients undergoing elective abdominoplasty at the Department of Plastic Surgery, Hand Surgery–Burn Center at the University Hospital RWTH Aachen were recruited for the study. Informed consent was obtained by all patients. The study protocol was approved by the regional ethics committee (ethics committee of the RWTH Aachen University Faculty of Medicine, Aachen, Germany; EK163/07), and the reported investigations have been carried out following principles endorsed by the Declaration of Helsinki.

Adipose tissue samples used for isolation of ASCs were processed as described earlier [15]. Adipose tissue was minced and digested in collagenase solution (0.2% collagenase I in PBS) for 45 min at 37 °C. Digested tissue was filtered through a 250 nm nylon mesh (Neolab, Heidelberg, BW, Germany). Oil and cell debris as well as the mature adipocyte fraction were separated from the stromal vascular fraction (SVF) by centrifugation at 400 xg for 10 min. Afterwards, the SVF was resuspended in proliferation medium (DMEM, 10% FBS), seeded on culture plates and cultured at 37 °C, 5% CO_2_. From these primary cultures, experiments were conducted with cells from passages 1–4 seeded at a density of 3 × 10^4^ cells per cm^2^.

### Pharmacological stimulation

Because THC is characterized by high lipophilicity, it has to be diluted in an appropriate vehicle for application in aqueous solutions. Earlier studies investigating the effects of THC used non-aqueous solvents, emulsifiers or surfactant molecules, or mixtures of these agents as a vehicle. Therein, the surfactant molecule Kolliphor®EL (formally known as Cremophor EL) has been regularly applied as vehicle for better aqueous solubility and stability of THC [16]. However, we found that EL is not an inert vehicle but exerts a range of biological effects on ASCs, making it unsuitable for the planed experiments [17]. Therefore, we decided to use another surfactant, Kolliphor®P188 (also known as Poloxamer P188) does not affect ASC biology and has already been used as vehicle for THC solutions [18].

For a 1 mM solution, THC was dissolved from a stock solution in methanol into a vehicle solution consisting of saline with 7.5% methanol and 5% P188. Saline containing the respective concentration of methanol and P188 served as vehicle control (Veh). To ensure complete adherence of the cells, THC was freshly added to the media two days after seeding and during each media exchange.

### Cell viability

At the indicated time points, the cell viability was assessed by determining cell number using crystal violet (CV) staining and via measuring resazurin conversion by mitochondrial enzymes, which serves as an indicator of cellular metabolic activity. Each procedure followed the same regime as described earlier [13]. Cells were incubated with resazurin solution (final concentration 0.01 mg/mL in proliferation medium) for 45 min. Afterwards, fluorescence was measured at 590 nm (excitation = 544 nm) using a microplate reader (BMG Labtech, Ortenberg, HE, Germany). For CV staining, the cells were washed with PBS, fixed in 2-propanol followed by washing with 0.05% Tween20 in PBS. After staining the cells with 0.1% CV in aqua _bidest_, the adsorbed dye was washed out in 33% acetic acid. Absorbance was quantified at 595 nm.

### Cell differentiation

Adipogenic differentiation was induced by high glucose medium supplemented with 10% FBS and 10 µM insulin. Osteogenic differentiation medium was low glucose medium with 10% FBS, 0.25 µM dexamethasone, 10 mM β-glycerophosphate, 200 µM ascorbate-2-phosphate. Chondrogenic differentiation medium contained DMEM supplemented with 5 ng/mL TGF-β3, 1 μM dexamethasone, 50 µg/mL ascorbate-2-phosphate, 40 µg/mL proline, 90 µg/mL pyruvate, and 1 mL ITS 1 Premix (1 mg/mL insulin, 0.55 mg/mL transferrin, 0.67 μg/mL sodium selenite).The medium was replaced every 2 to 3 days. Evaluation of trilinear differentiation was performed as described earlier [13]: Adipogenic differentiation was assessed by Oil Red O staining. ASCs were PFA fixed and stained with Oil Red O solution (0.3% in isopropanol:aqua_bidest_, 3:2). Adsorbed dye was washed out with isopropanol and absorbance was measured at 540 nm. For evaluation of osteogenesis, PFA-fixed cells were incubated in cresolphthalein-buffer (50 mg o-cresolphthalein complexon and 500 mg 8-Hydroxyquinoline dissolved in 30 mL of 37% HCl, and diluted in 470 mL aqua_bidest_). After adding AMP buffer (76 mL AMP in 440 mL aqua_bidest_, pH = 10.7 with HCl), the extinction of the supernatant was quantified at 580 nm. Chondrogenic differentiation was measured by Safranin O staining. Differentiated cells were stained with 0.1% Safranin O aqua_bidest_. The adsorbed dye was washed out with isopropanol and quantified at 540 nm.

### Animals

Mice (C57BL/6 J; n = 18, female) were purchased from Janvier Labs (Tancon, France) and kept in the facilities of the Institute of Laboratory Animal Science at the University Hospital, RWTH Aachen. The mice were housed in groups of 4–5 animals in plastic cages with sawdust bedding and had access to food and water ad libitum. The animal housing rooms were kept at 21–24 °C and 40–60% relative humidity with a 12-h light/dark cycle. The animals were randomly divided into two groups and treated with either THC or vehicle solution, respectively, over a time course of 21 days. After daily determination of the bodyweight (BW), the animals received an injection (200 µL, *s.c.*) of either THC (3 mg/kg BW), or vehicle solution [16]. Mice were sacrificed for experimental investigation by cervical dislocation in deep anesthesia (5% isoflurane). The skin was disinfected with 70% ethanol, and adipose tissue was obtained from fully excised perigonadal and perirenal fat deposits for determination of growth factor contents as well as for histological analysis [12]. The animal experiments were performed according to EU Directive 2010/63/EU for animal experiments, they followed the guidelines of the animal welfare laws and were approved by the Animal Care and Use Committee of the state of North Rhine-Westphalia, Germany (AZ-81–02.04.2021.A352).

### Histology

Adipose tissue of mice was cut into samples of ~ 2–3 mm side length, fixed in 4% PFA for 24 h and then embedded in paraffin. Slices of 2 µm thickness were prepared on a microtome (Hyrax M40, Zeiss, Oberkochen, BW, Germany), mounted on microscope glass slides and dried overnight in an incubator (37 °C). After dehydration in an ascending alcohol series and xylene, the slides were cover slipped with ROTI®Histokitt (Carl Roth, Karlsruhe, BW, Germany). In each slide, 3–5 randomly chosen regions of interest (ROI = 290 × 228 µm^2^) were photographed on an EVOS FL auto imaging system (Thermo Fisher Scientific, Waltham, MA, USA). The volume of lipid vacuoles was determined as indicator of fat cell size using the free software ImageJ (Wayne Rasband, Institutes of Health, Bethesda, Rockville, MD, USA).

### Cytokine determination by enzyme-linked immunosorbent assay (ELISA)

After seven days of stimulus exposure (Veh or THC), the cell supernatant of each well from the viability experiments was collected to determine the concentrations of the hepatocyte growth factor (HGF), the transforming growth factor (TGF)‐β1 and the vascular endothelial growth factor (VEGF), by enzyme-linked immunosorbent assay ELISA Duo‐Sets (R&D Systems, Minneapolis, MN, USA) following the manufacturer's instructions. Extinction was measured in duplicates per well. Cytokine concentrations were expressed as the amount of each factor (in ng) per number of cells (OD crystal violet).

For the evaluation of cytokine content in adipose tissue *in vivo*, the concentrations of HGF, TGF-β1 and VEGF were determined. The adipose tissue was homogenized in 2 mL lysis buffer (pH = 7.5, 10 mM HEPES, 0.5% Triton X-100, protease inhibitor) on ice using an Ultra-Turrax tissue homogenizer (IKA Works, Inc, Wilmington, NC, USA). The homogenized suspension was first centrifuged at 1,000 xg for 10 min at 4 °C, followed by second centrifugation at 14,000 xg at 4 °C for 40 min. The oily phase and cell pellet (consisting of non-homogenized cells and nuclei) were discarded. The supernatant was divided into aliquots and taken for cytokine determination as described above. Cytokine concentrations were expressed as the amount of each factor per mg of soluble extracted protein. Whole protein concentrations of the probes were assessed by the DC Protein Assay (BioRad, Hercules, CA, USA) as specified by the manufacturer.

### Statistical analysis

Data of all experiments were grouped to evaluate the results for each type of experiment and were tested for normal distribution using the Kolmogorov–Smirnov test. Normally distributed data were presented as mean values (+ SEM) and were statistically analyzed using Student’s t-test or by analysis of variance (one-way or two-way ANOVA) followed by pairwise comparison using the Dunnett post-hoc test for THC-exposure *vs.* Veh. Non-normally distributed data were presented as box-plots (median as the middle line and 25/75% as box boundaries) and were statistically analyzed by Mann–Whitney U-test or by Kruskal–Wallis H-test followed by pairwise comparison using the Mann–Whitney U-test after Bonferroni correction (SPSS 24, SPSS Inc., Chicago, IL, USA). Differences associated with *p* ≤ 0.05 were considered statistically significant. Figures were created using the graphics program Corel Draw X5 (Corel Corporation, Ottawa, ON, Canada).

## Results

### Effects of THC on ASC proliferation and metabolic activity

**Fig. 1:**
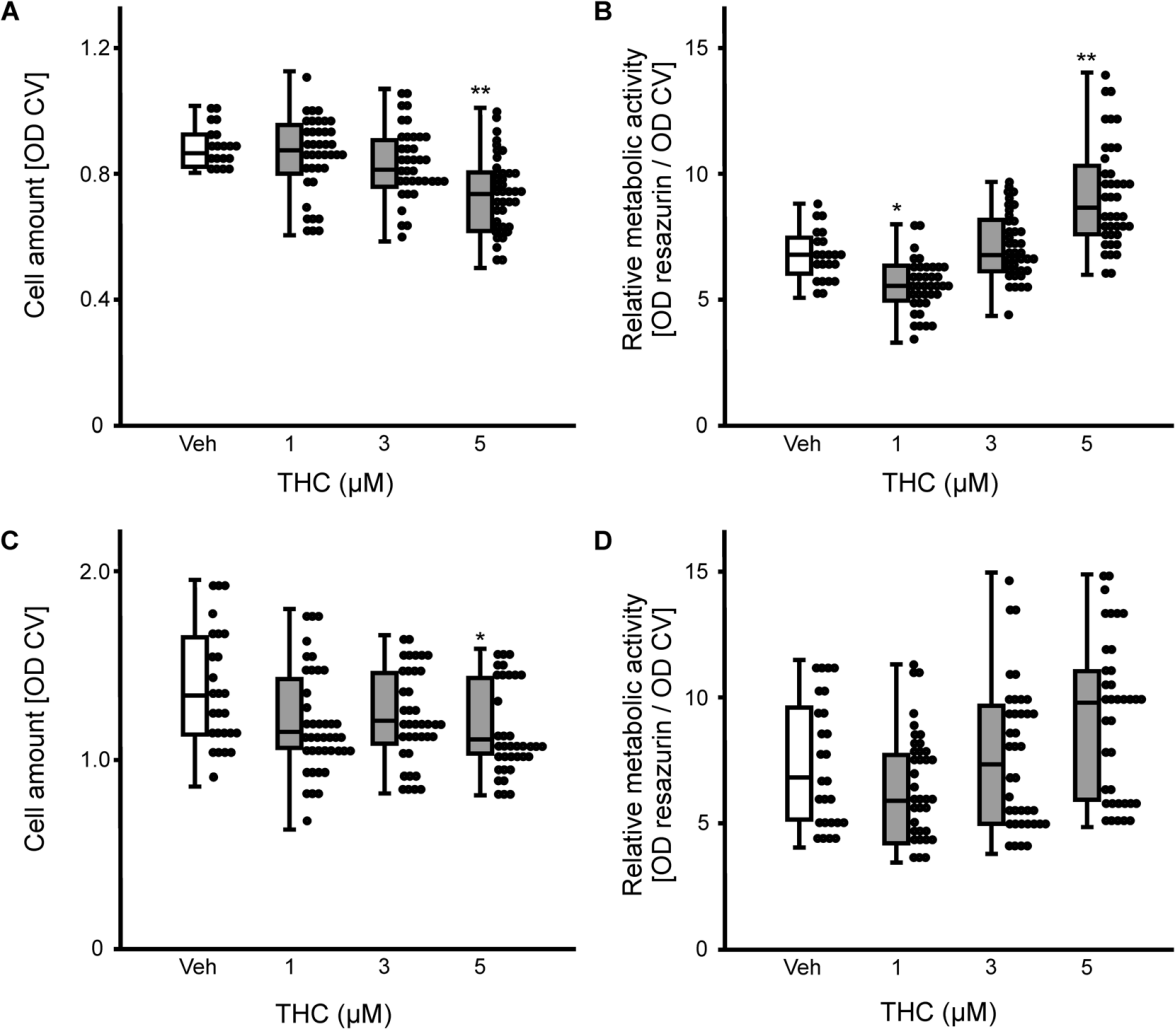
Influence of THC application (1–5 µM) on the cell amount and the metabolic activity of ASCs after. **A** 7 days and **B** 14 days of incubation. Cell numbers were assessed by optical density (OD) of crystal violet (CV) staining, metabolic activity was determined by resazurin conversion. Box plots represent medians calculated from optical densities with the data points in a scatter plot. The number of experiments and donors were n ≥ 24 (3 donors). Statistical analyses were performed by Kruskal–Wallis H-test followed by pairwise comparison *versus* the vehicle control (Veh); *p ≤ 0.05, and **p ≤ 0.01

ASCs were exposed to increasing THC-concentrations (1–5 µM). Proliferation was assessed by determining absolute cell numbers using crystal violet staining, and metabolic activity per cell was evaluated by resazurin conversion. Kruskal–Wallis H-test determined significant differences between the types of treatment for the cell numbers (H(3) = 23.42, *p* ≤ 0.001) and for the metabolic activity (H(3) = 62.96, *p* ≤ 0.001) after 7 days. Post-hoc analysis found that the highest dose of THC tested in the experiments (5 µM) decreased proliferation of ASCs (*p* = 0.001; Fig. 1A), but increased metabolic activity (*p* = 0.001; Fig. 1B). By contrast, 1 µM THC decreased significantly metabolic activity after 7 days (*p* = 0.023). H-test determined significant differences between the treatments for cell numbers (F(3) = 8.53, *p* = 0.036) and for the metabolic activity (F(3) = 17.04, *p* ≤ 0.001) after 14 days. Again, as observed for the first time point of investigation, 5 µM THC decreased cell number (*p* = 0.027; Fig. 1C), however, pairwise comparison failed to detect a difference for metabolic activity per cell after 14 days of exposure (Fig. 1D).

### Effects of THC on differentiation of ASCs

**Fig. 2:**
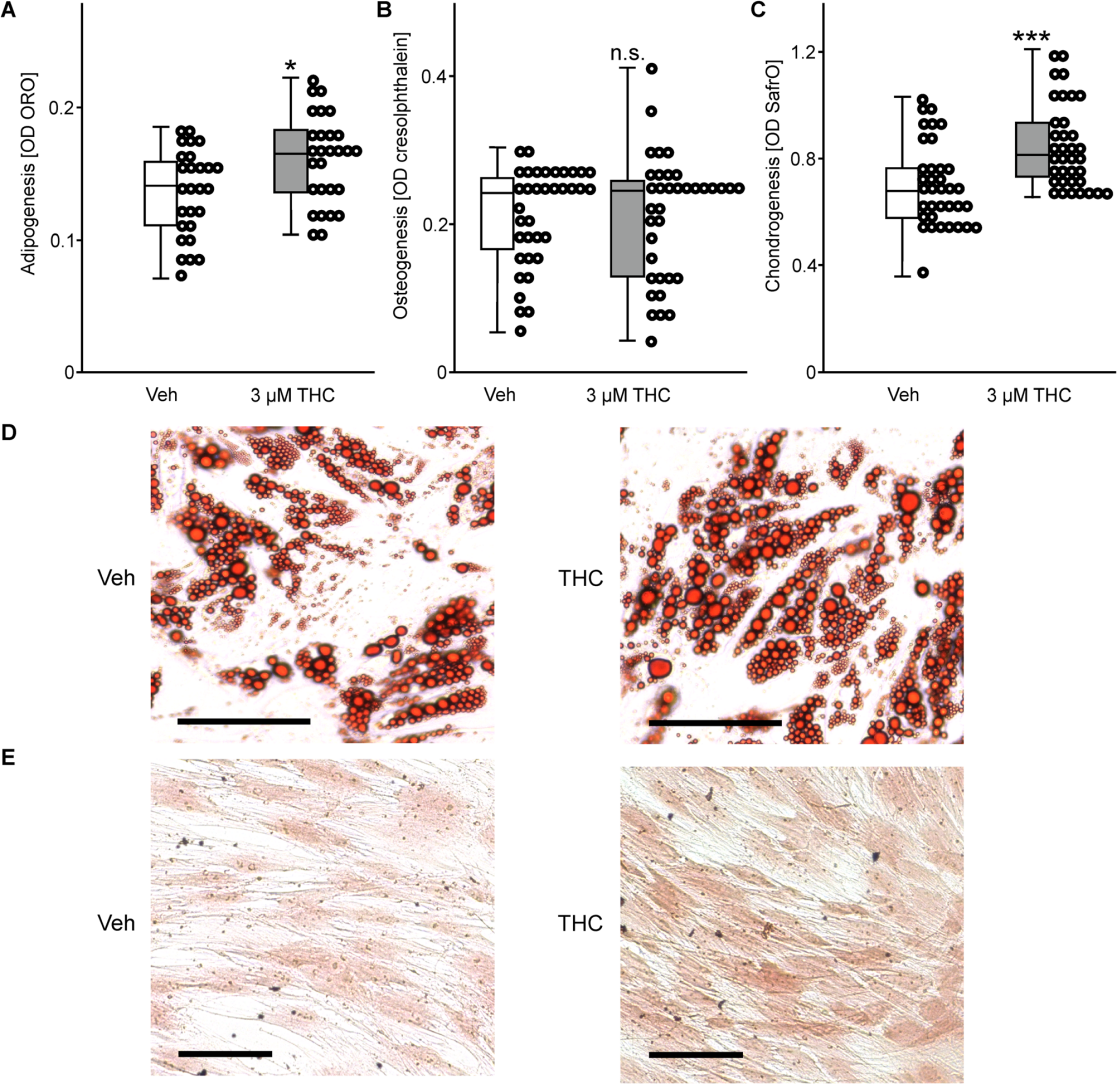
Effect of THC exposure on trilinear differentiation of ASCs. Adipogenesis was measured after 14 days by **A** Oil Red O staining. Osteogenesis was analyzed by **B** cresolphthalein staining for quantification of the extracellular matrix calcification after a 14-day incubation period. After 5 days of stimulation, chondrogenesis was quantified by **C** Safranin O staining. Photographs indicate differences in the number and size of **D** stained lipid droplets in adipocytes (bar = 50 µm), and **E** proteoglycan formation in stained chondrocytes (bar = 100 µm). Box plots represent medians calculated from optical densities with the data points in a scatter plot. The number of experiments and donors was as follows: **A** n = 27 (3 donors); **B** n = 36 (3 donors); **C** n = 36 (4 donors). Statistical analysis was performed using the Mann–Witney U-test: *p ≤ 0.05, and ***p ≤ 0.001

The effects of THC exposure (3 µM) on trilinear differentiation of ASCs cultured in specific differentiation medium was analyzed by colorimetric measures after 14 days. Adipogenic differentiation was evaluated by lipid staining, which was increased upon THC-treatment (t(53) = 2.86, *p* = 0.06; Fig. 2A, D). To measure the effect of THC on osteogenic differentiation, the mineralization of the ASC extracellular matrix was determined by cresolphthalein staining, which was not affected upon stimulus exposure (t(70) = 0.08, *p* = 0.94; Fig. 2B). After 5 days of stimulation, chondrogenic differentiation was analyzed by Safranin O staining. This dye labels glycosaminoglycans in developing or mature chondrocytes, which was significantly increased upon THC exposure (t(70) = 3.95, *p* ≤ 0.001; Fig. 2C, E).

### Effects of THC on growth factor release by ASCs—*in vitro*

**Fig. 3:**
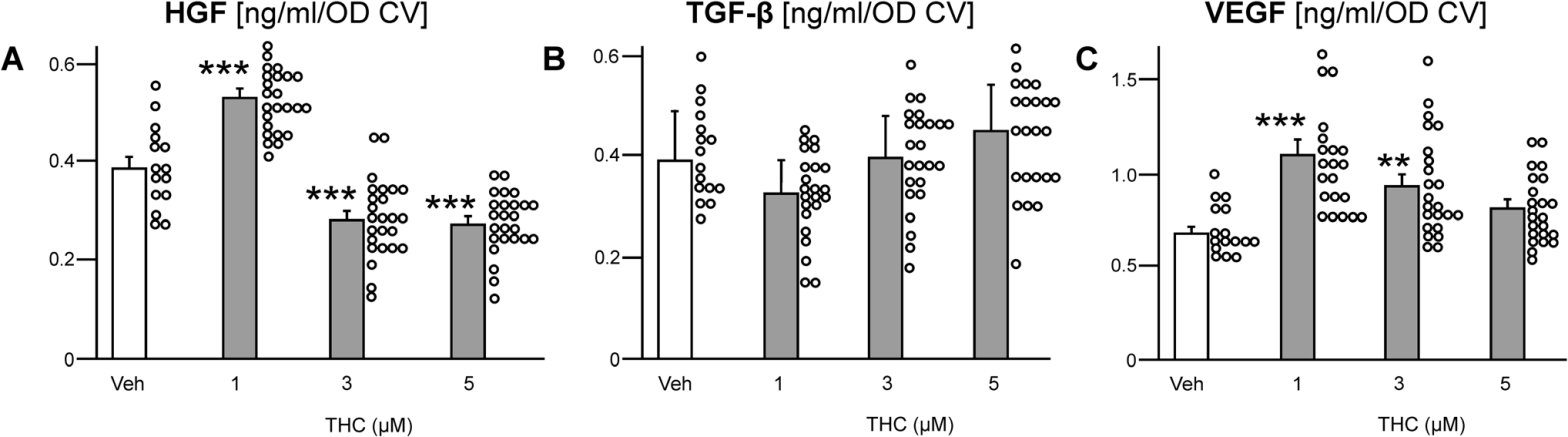
Influence of THC stimulation on growth factor release by ASCs. Cells were stimulated with THC at increasing concentrations (1–5 μM). After 7 days of treatment, media were collected to determine growth factor secretion by sandwich ELISA: **A** HGF, **B** TGF-β1, **C** VEGF. The concentration of each factor is presented as ng/mL per cell number [OD CV]. Data are expressed as mean values (+ SEM) with the data points in a scatter plot. The number of experiments and donors were n ≥ 15 (3 donors). Statistical analyses were performed by one-way ANOVA followed by pairwise comparison *vs.* the vehicle control (Veh); **p ≤ 0.01, and ***p ≤ 0.001

The distribution of the growth factors release by ASCs after 7 days of pharmacological stimulation is summarized in Fig. 3. One-way ANOVA determined significant differences between treatment for HGF concentration (F(3, 86) = 63.25, *p* ≤ 0.001). Dunnett`s post-hoc test found that THC increased HGF release at 1 µM (*p* < 0.001; Fig. 3A), but when applied at higher concentrations (≥ 3 µM) it also decreased the HGF content in the cell supernatant (*p* < 0.001). By contrast, low concentration of THC (< 3 µM) decreased TGF-β1 release, whereas high concentration (> 3 µM) increased TGF-β1 release by ASCs (Fig. 3B). However, the differences were not significantly different from the Veh-control. THC affected also the secretion of VEGF (F(3, 79) = 9.8, *p* ≤ 0.001). There was an inverse concentration-dependent effect, *i.e.*, THC at 1 µM (*p* ≤ 0.001) and at 3 µM (*p* = 0.008) increased VEGF release (Fig. 3C), while 5 µM of THC did not produce an effect.

### Effects of THC treatment on adipocyte size, bodyweight, and growth factor content in adipose tissue—*in vivo*

**Fig. 4:**
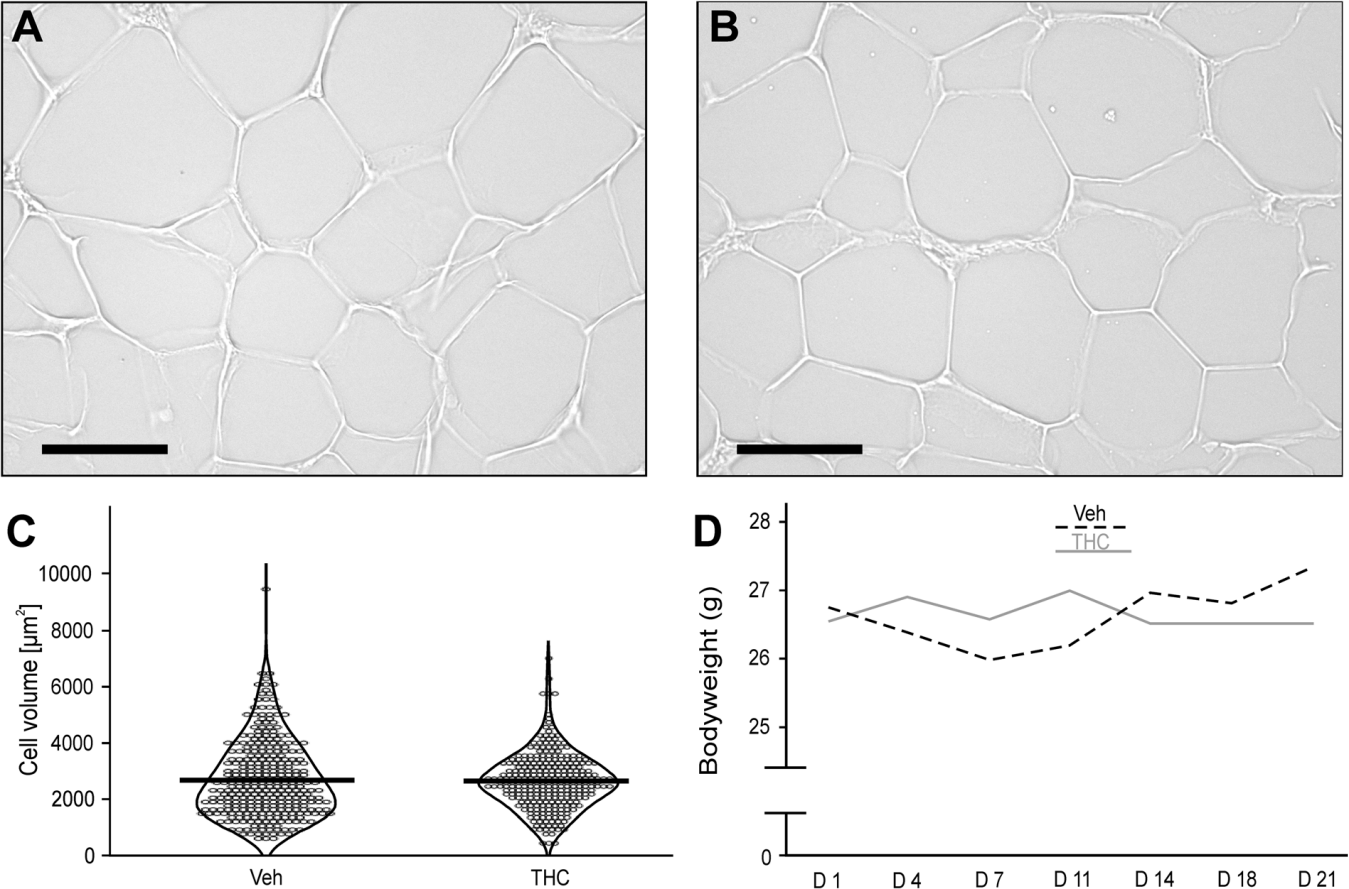
Representative photographs of adipocytes from a mouse of **A** the vehicle control group (Veh) and **B** the THC cohort (3 mg/kg). The volume of adipocytes was determined by measuring the area of lipid vacuoles (bar = 50 µm). The cell sizes of 9 animals per group (Veh: n = 319 cells, THC: n = 284 cells) is presented in **C**, data points are presented in violin plots, thick black line indicates the mean value. The development of the mean bodyweight of experimental animals over the time course of substance application is shown in D

**Fig. 5:**
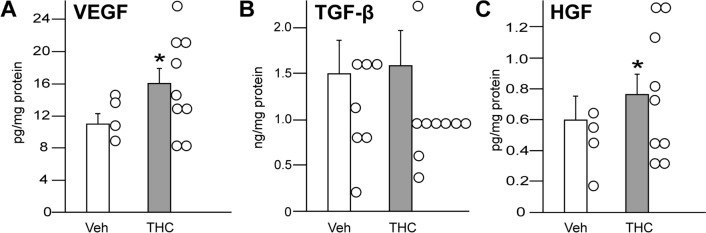
Influence of THC treatment on growth factor concentration in adipose tissue of mice. Animals received a daily injection (*s.c.*) of either vehicle (Veh) or THC solution (3 mg/kg BW). After 21 days, adipose tissue was collected to determine growth factor content by sandwich ELISA: **A** VEGF, **B** TGF-β1, **C** HGF. The concentration of each factor is indicated per mg of the whole protein amount. Data are expressed as mean values (+ SEM) with the data points in a scatter plot. The number of animals were n = 9 per group. Statistical analysis was performed by Student’s t-test; *p ≤ 0.05

During the process of adipogenesis, the cells, *i.e.*, ASCs and adipocytes, accumulate cytoplasmic triglycerides, which increases their volume. Morphometric analysis revealed that each animal presented a broad distribution of fat cell sizes (Fig. 4A, B). Mean adipocyte volume did not differ in mice treated with the vehicle (2673 μm^2^ SEM ± 75 μm^2^) when compared to animals that received the THC-solution (2624 μm^2^ SEM ± 59 μm^2^; U = 0.78, *p* = 0.434; Fig. 4C). As the chronic THC-application did not affect the cell size in adipose tissue, this treatment had also no impact on the bodyweight of the animals. During the time course of the pharmacological treatment, the mean-bodyweight of the vehicle group was between 26.0 and 27.4 g, which was not different from the THC cohort ranging between 26.5 and 27.0 g (F(1, 15) = 0.02, *p* = 0.885; Fig. 4D). The daily injection of THC increased the concentration of VEGF (t(10.8) = 2.44, *p* = 0.033; Fig. 5A) in the adipose tissue of experimental animals. While this procedure did not affect the content of TGF-β1 (t(15) = 1.06, *p* = 0.31; Fig. 5B), it increased the amount of HGF ((t(11.66) = 2.57, *p* = 0.025; Fig. 5C).

## Discussion

Adipose tissue is present in large quantity and represents an ideal autologous material for tissue augmentation, as well as for reconstruction and rejuvenation purposes. For example, lipofilling has been applied for total breast reconstruction after mastectomy [19], for functional reconstruction of the sole of the foot [20], and for therapy of burn wound healing and scarring [21]. Although ASCs are not the only cellular component involved in tissue regeneration and graft survival, these cells are attributed as key factors because of their ability for proliferation and differentiation, and their secretory activity [22]. Because the ECS modulates stem cell fate, e.g., proliferation and differentiation, as well as the composition of the cells` secretome [23], we tested if the treatment with a chronic low dose of (medical) THC would prime ASCs for enhanced regenerative properties.

We found that ASCs tolerated exposure to concentrations of ≤ 3 µM THC, while treatment with a higher dose decreased the cell numbers. This is in accordance with an earlier study, which reported that stimulation with 1 nM–0.1 µM of THC for 48 h does not affect numbers of 3T3-L1 preadipocytes, whereas 500 nM increases proliferation in a CB2 dependent mechanism [24]. However, this study did not test higher concentrations of THC for its effect on proliferation. Our data have confirmed that stimulation of CB2 with low concentrations of the specific receptor agonist JWH-133 increases ASC proliferation, while we found also that co-activating CB1 and CB2 using WIN55,212-2 abolishes this effect [14]. This suggests a bi-phasic effect of CB1 and CB2 interaction on cell proliferation, which is concentration-specific of the specific receptor ligand. Because we did not test the effect of 0.5 µM THC but of 1 µM, this could explain the absence of a pro-proliferative effect in the present study. Furthermore, the inhibition of cell proliferation has also been validated for mesenchymal stem cells from the bone marrow at exposure to 5 µM THC, which complies with the present findings [25]. Numerous studies report the cytotoxic and cell growth inhibitory effects of phytocannabinoids, including THC, on cancer and cancer stem cells [26]. The interaction of THC with the cannabinoid receptors CB1 and CB2 can inhibit cell proliferation, e.g. by inducing cell cycle arrest via downregulation of the E2F1 transcription factor [27]. Since cancer stem cells share many common characteristics with somatic stem cells, it is possible that the same molecular processes are responsible for the findings of the present study on ASCs [28]. The ECS represents a key factor in regulating ASC and adipocyte metabolism on various levels, as it has been shown that CB1-agonists produce insulin-mimetic effects by increasing cellular glucose uptake via enhanced activity of GLUT4 and PI3-kinase [29]. Elevated glucose and oxygen consumption characterize a metabolic switch from glycolysis to oxidative metabolism, *i.e.*, mitochondrial activity [30]. THC at 5 µM increased the metabolic activity of ASCs assessed by resazurin conversion, which is based on the activity of mitochondrial enzymes in the respiratory chain [31]. This result is comparable to the effect of anandamide signaling, since this endocannabinoid also increases metabolic activity of ASCs [15].

Cannabinoids, including THC, bind to various receptors that are expressed on the cell membrane or on intracellular structures and participate in different physiological functions targeting cell differentiation. THC binds with high affinity to CB1 [32], which stimulates glucose uptake and increases metabolic activity. Both events characterize the switch from glycolysis of quiescent cells to high mitochondrial metabolism of adipogenically differentiating ASCs [30]. In addition, THC has high affinity to peroxisome proliferator-activated receptor (PPAR)-γ [33]. Amongst all pro-adipogenic transcription factors that have been characterized so far, PPAR-γ is regarded the master regulator of adipogenesis [34]. As a result, THC exposure increases the differentiation of ASCs and preadipocytes into fat-storing, functional adipocytes [24]. On the other hand, THC treatment did not affect osteogenic differentiation of ASCs, although it has been reported that cannabinoids support the development into osteoblasts [15]. The endocannabinoid anandamide binds to the G protein-coupled receptor (GPR)55 [32], which stimulates Ca^2+^-influx that is important for osteogenesis in ASCs [35]. Accordingly, we have found that blocking GPR55 inhibits the pro-osteogenic effect of anandamide [13]. The absent effect of THC on ASC osteogenesis could be dependent on interaction with other receptors that probably superimpose the impact of THC on GPR55. Finally, THC supported chondrogenic differentiation of ASCs by increasing proteoglycan deposition, which is similar to the effect of WIN55,212-2 [14]. Likewise, mesenchymal stem cells from the bone marrow display enhanced collagen RNA and protein expression as well as increased Safranin O staining of proteoglycans upon THC exposure [36]. However, the exact mechanism or pathway how cannabinoids affect chondrogenic differentiation requires further clarification.

Stimulating the ECS with cannabinergic compounds, either endogen, herbal or synthetic, is well known for its immunomodulatory effectivity [37]. While some experimental investigations have been performed *in vivo* [38, 39], the majority of the studies tested the impact of cannabinoids on immune cells *in vitro*. THC down-regulates especially the secretion of IL-6 [40], whereas it is less effective on the release of IL-1β, TNF-α and MCP-1 [41]. Since the immune-suppressive activity of cannabinoids is thoroughly approved, their pro-regenerative capacity has been less intensively explored. We found that stimulating ASCs with endocannabinoids or with specific agonists of CB1 and CB2 receptors increases the release of regenerative growth factors, *i.e.*, VEGF, HGF, TGF-β1, and IGF [14, 15]. In accordance with these earlier studies, we now found that also THC increased the release of VEGF and HGF, which are two important factors promoting blood vessel formation in the developing adipose tissue [42]. This result highlights the undoubted ability of cannabinergic compounds to modulate vascularization, which opens a new therapeutical route for angiogenesis-related problems or pathologies, e.g. fat grafting [43].

Knowledge of the pharmacokinetic profile of administered THC is important to assess its availability in the body. In addition, the route of drug administration and drug formulation determine the rate of drug absorption. A number of human studies have investigated the disposition of THC and its metabolites after smoking, which is the principal route of cannabis administration [44]. On the other hand, fewer investigations analyzed oral administration that, however, is the recommended route for therapeutic applications. Approved cannabinoid substances are Sativex, the medicinal THC (∆^9^-tetrahydrocannabinol) that is an under‐the‐tongue spray, as well as its synthetic forms, e.g., nabilone, dronabinol, and marinol [45].

In general, absorption is slow when cannabinoids are ingested, with a low and delayed (1–5 h) peak THC concentration in plasma [46]. Admittedly, the dose, vehicle, and individual physiological factors such as rates of metabolism and excretion also influence THC concentrations in circulation. In mice, the peak THC concentration in plasma has been observed at 1 h after oral administration (30 mg/kg BW), while it is still detectable after 24 h [47]. On the other hand, when THC is applied by *i.p.* injection, the plasma peak occurs after 15–30 min [48]. A comparable time course occurs for the THC distribution in the brain, where the highest THC concentration has been determined after 60–120 min, followed by a decline during the subsequent 4–8 h. By contrast, THC also peaks in adipose tissue after 30–120 min, but the concentration does not decrease afterwards. Thus, adipose tissue, *i.e.*, adipocytes, can store this hydrophobic substance for long time and can release it upon stimulation [49]. A chronic low dose of THC (by daily *s.c.* injection) could have led to accumulating and perpetuating THC concentrations in adipose tissue of the experimental animals. Therein, the continuous exposure to THC could have been too short in time or too low in concentration to affect adipocyte size or adipogenesis, as also the bodyweight of the mice did not differ between the experimental and the control group. Whereas the THC concentration might have been sufficient for potentially stimulation of the local ASCs to release increased amounts of growth factors, e.g. HGF and VEGF. We therefore argue that the described paradigm of mildly stimulating the ECS of ASCs *in vivo* could improve the regenerative potential of adipose tissue, for example when being applied for lipofilling.

ASCs contribute significantly to the therapeutic potential of adipose tissue, because these cells have the multipotent differentiation capacity and they release regenerative soluble factors. The present study is the first to investigate the effect of stimulating an endogen modulatory system expressed by ASCs on their regenerative capacity. Exposing ASCs to a chronic low dose of THC promotes differentiation into adipocytes and chondrocytes, but not osteoblasts, and it increases the release of the pro-angiogenic growth factors HGF and VEGF. For the *in vivo* transfer, mice received a daily injection of THC for 3 weeks, which did not affect adipogenic development but increased the concentration of HGF and VEGF in adipose tissue samples. Our findings may offer a new approach for improved application of adipose tissue in regenerative medicine.

## Data Availability

The datasets analyzed during the current study are available from the corresponding author on reasonable request.
